# Choroidal Vascularity Index Changes After Exercise in Patients With Glaucoma

**DOI:** 10.3389/fphys.2022.844795

**Published:** 2022-03-30

**Authors:** Dan Cheng, Jia Fang, Weiqian Gao, Minhui Wu, Yilin Qiao, Kaiming Ruan, Hengli Lian, Jiner Cen, Lin Fu, Lijun Shen, Li Nie

**Affiliations:** ^1^ The Affiliated Eye Hospital of Wenzhou Medical University, Hangzhou, China; ^2^ Yongkang First People’s Hospital of Hangzhou Medical College, Yongkang, China

**Keywords:** choroidal vascularity index, exercise, glaucoma, optical coherence tomography, primary open-angle glaucoma

## Abstract

**Purpose:** To investigate the changes in choroidal vascular structures after exercise in patients with glaucoma using an image binarisation algorithm with enhanced-depth imaging optical coherence tomography.

**Methods:** Thirty-four eyes of 19 patients with primary open-angle glaucoma and 40 eyes of 20 normal subjects were included in the glaucoma and control groups, respectively. All subjects were evaluated before, immediately after, and 30 min after 20-min running at moderate speed. The subfoveal choroidal area was segmented into the luminal area (LA) and stromal area (SA), and the choroidal vascularity index (CVI) was measured by calculating the ratio of LA to the total choroidal area (TCA). The mean intraocular pressure (IOP), subfoveal choroidal thickness, CVI, SA, CA, and TCA were compared within and between both groups. The correlation between CVI, IOP, and other vascular indexes was investigated.

**Results:** In the glaucoma group, a significantly lower CVI was found immediately after exercise and recovered 30 min after exercise. Higher TCA and LA levels were demonstrated 30 min after exercise compared to immediately after exercise. In both groups, IOP decreased immediately after exercise but was restored after a 30-min rest. In the glaucoma group, there was a higher correlation between CVI and other choroidal vascular parameters than in the control group. There was no difference in IOP or choroidal parameters between the groups at different time points.

**Conclusion:** In patients with glaucoma, CVI decreased significantly immediately after exercise, indicating that the choroidal layer is affected by exercise and an unhealthy vascular regulatory mechanism.

## Introduction

Glaucoma is one of the leading causes of irreversible vision loss and is characterised by accelerated apoptosis of retinal ganglion cells (Flaxman et al., 2017). Vascular factors causing fluctuation and reduction in ocular blood flow (OBF) contribute to neurodegeneration in glaucoma. Pinto et al. found that patients with glaucoma had lower retrobulbar velocities compared to normal controls (Abegão Pinto et al., 2016). Moreover, glaucoma affects not only the retinal circulation but also the choroidal vasculature. Portmann et al. showed that in patients with primary open-angle glaucoma (POAG) and ocular hypertension, the choroidal blood flow was lower compared to the normal controls (Portmann et al., 2011). The involvement of the choroidal vasculature in patients with glaucoma remains controversial (Banitt et al., 2013).

Moreover, knowledge concerning the changes that occur in the choroidal vasculature in patients with glaucoma after exercise is limited. Recently, some published studies have focused on the effect of physical exercise on intraocular pressure (IOP) and OBF and demonstrated the benefits of exercise in delaying the progression of glaucoma (Zhu et al., 2018). However, previous studies have shown that the OBF remains constant despite ocular perfusion pressure (OPP) variation owing to the mechanism of autoregulation (Khayi et al., 2011; Risner et al., 2009). In contrast, an existing evidence has opposed the theory of autoregulation in the choroidal vessels during dynamic exercise (Hayashi et al., 2011). Furthermore, in patients with glaucoma, the autoregulation of choroidal circulation is weakened due to pathological changes (Portmann et al., 2011).

With the development of spectral-domain optical coherence tomography (OCT) technology, the choroidal vasculature can be quantified *in vivo* (Agrawal et al., 2020; Park et al., 2018). Enhanced-depth imaging (EDI)-OCT enhances the visualization of the choroidal scleral interface and allows quantitative assessment of choroidal thickness (CT). More recently, a novel quantitative imaging biomarker known as the choroidal vascularity index (CVI) has been proven to be a potential tool for establishing early diagnoses, monitoring disease progression, and prognosticating patients {Agrawal et al., 2020}. CVI was a new metric to assess vascularity of the choroid based on OCT. {Agrawal, 2016} The area of dark pixels within the choroidal area was determined as the luminal area (LA). CVI was computed by dividing LA by the total subfoveal circumscribed choroidal area (TCA). Compared to CT, which may be associated with multiple patient factors, including age, axial length, IOP, and systolic blood pressure, CVI is robust and relatively resistant to changes in physiological parameters. Thus, there is a need to explore the use of CVI as a stable marker for the robust assessment of choroidal health.

The current study aimed to characterize the CT and vascularity index and the changes that occurred after exercise in patients with glaucoma using EDI-OCT. In addition, we investigated the differences in choroidal parameters between patients with glaucoma and normal controls.

## Materials and Methods

This study was approved by the Ethics Committee of Wenzhou Medical University. This study was conducted in accordance with the Declaration of Helsinki, and informed consent was obtained from all the patients. In this prospective study, 19 patients with POAG and 20 healthy, age- and sex-matched control subjects between april 2020 and February 2021 were included from the outpatient clinic of the Department of Glaucoma at the Eye Hospital, Wenzhou Medical University.

The inclusion criteria were as follows: patients with present visual acuity of 16/20 or higher, with a spherical equivalent refractive error greater than −8.0, with astigmatism within 2 diopters, and with age range of 18–45 years. The exclusion criteria were as follows: patients with exfoliation glaucoma and pigmentary glaucoma, with a history of acute angle closure, with a mean deviation of visual field testing (Humphrey visual field analyzer, 30–2 with near correction, SITA pac) worse than −20 dB, with IOP exceeding 21 mmHg controlled with drugs, with ocular inflammation or infection within the last 3 months, and undergoing intraocular surgery or laser trabeculoplasty within the last 6 months and pregnant patients. The control group comprised healthy subjects who were age-, sex-, and spherical equivalence-matched; had no ocular disease or systemic disease, such as diabetes or hypertension; and were not pregnant or smokers or subjects with no history of ocular surgery.

Each subject underwent a screening examination, including medical history, and a complete ocular examination, including best-corrected visual acuity measurement (measured by a Snellen chart), IOP measurement (Goldmann applanation tonometry), slit-lamp biomicroscopy, and gonioscopy. Axial length was measured using an Intra Ocular Lens Master (Carl Zeiss Meditec, Germany), and visual field examinations were performed by Humphrey perimetry. Spectral-domain OCT (SD-OCT, Heidelberg Engineering, Heidelberg, Germany) was used to evaluate the retinal nerve fiber layer (RNFL). The diagnostic criteria for POAG were as follows: pathological optic disc appearance, glaucoma hemifield test outside normal limits, and pathological thinning of RNFL shown on OCT.

Physical exertion consisted of 20 min of continuous running at a uniform speed on a treadmill. To simulate moderate-intensity daily exercise, the speed ranged from 6 to 8 km/h. Exercise was discontinued if the subject experienced intense discomfort or dyspnoea. OCT and IOP were repeated immediately after running (without any recovery period) and after a 30-min rest. Two experts analyzed the data. A third expert (DC) settled the situation in the case of a disagreement.

### Enhanced-Depth Imaging-Optical Coherence Tomography

**FIGURE 1:**
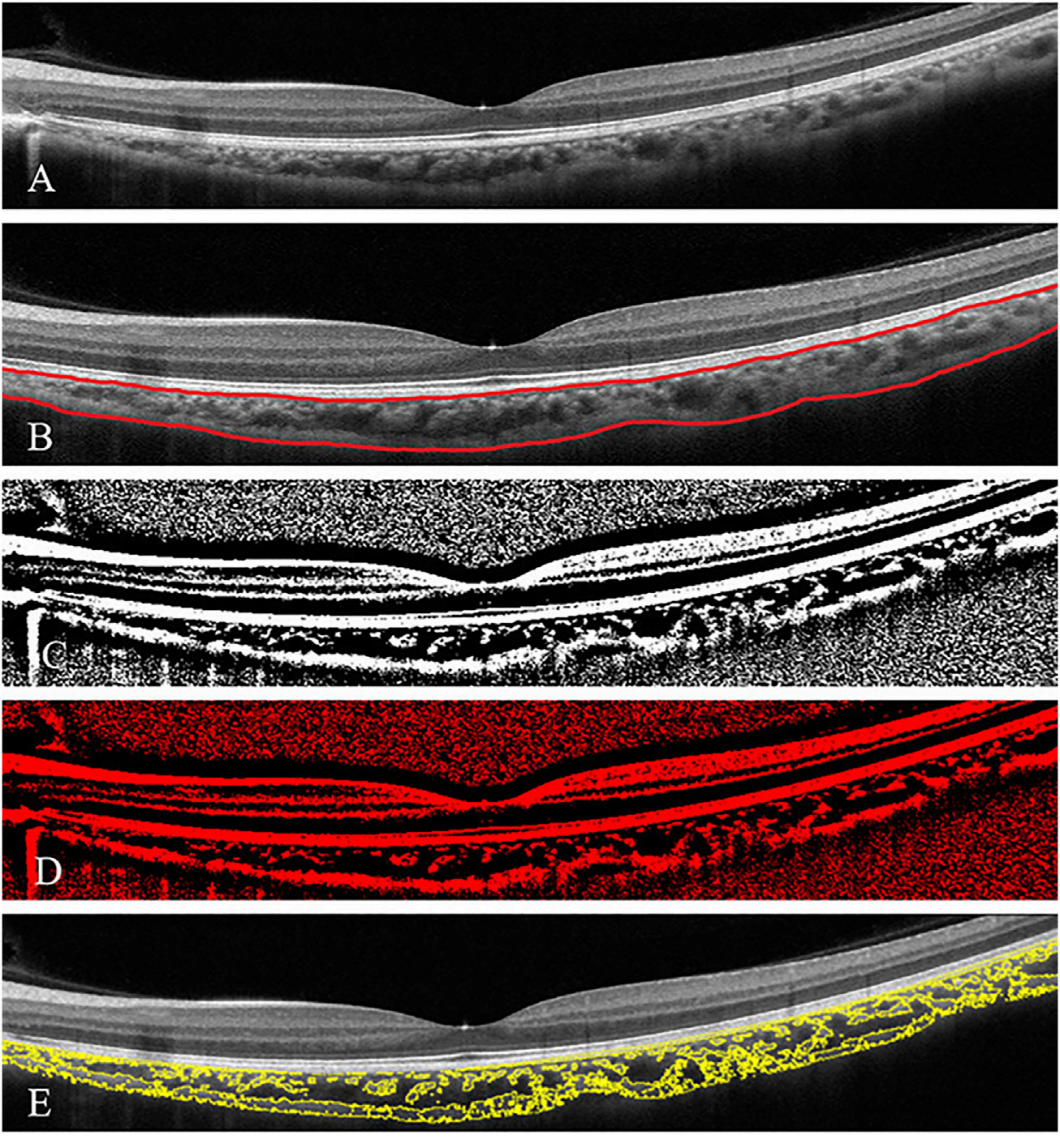
Subfoveal choroidal thickness and choroidal vascularity index (CVI) analysis in a patient with glaucoma **(A)**. The enhanced-depth imaging optical coherence tomography (EDI-OCT) image of one eye of patient with glaucoma **(B)** Choroidal boundaries were automatically identified and assessed by the two operators (red lines) **(C)** The total choroidal area (TCA) was determined by the binarised EDI-OCT image **(D,E)** CVI was derived as a proportion of the choroidal vasculature (yellow outlines) against the TCA by image binarisation.

EDI-OCT horizontal single line scan (Heidelberg Engineering, Heidelberg, Germany) encompassing the fovea was taken from patients and controls. To limit the effect of circadian rhythm on choroidal parameters (Usui et al., 2012), EDI-OCT images were collected between 9:00 AM and 12:00 AM. Two different evaluations of the choroid, CT analysis, and CVI calculation were performed. Subfoveal CT (SCT) was defined as the vertical distance between the outer surface of the retinal pigment epithelium (RPE) and the choroidal-scleral interface underneath the fovea and was assessed by two independent skilled operators (JF and KL) with the OCT software (Eye Explorer version 6.15.7.0, Heidelberg Engineering) embedded in the software (Figure 1.B). Interobserver agreement was calculated, and the average of measurements from the two operators was used for the analysis.

The macular SD-OCT scans were binarised using the public domain software ImageJ 1.51s (National Institutes of Health, Bethesda, MD, United States). The polygon tool was used to assess the region of the choroid area across the entire length of the EDI-OCT image. Furthermore, the image was converted to 8 bits, and autolocal thresholding (Niblack method) was performed on the binarised image. The image was again converted to red, green, and blue to select dark pixels representing the luminal area (LA), defined as the vascular area of the choroid. The subtraction of LA from the total choroidal area (TCA) was computed as the stromal area (SA). The CVI was calculated by dividing the LA by the TCA, defined as the area between the RPE and the choroid scleral junction (Figure 1) (Agrawal et al., 2020).

### Statistical Analysis

Statistical analysis was performed using the Statistical Package for the Social Sciences statistical software (SPSS Inc., Chicago, IL, United States). For normal distribution, data are expressed as the mean ± standard deviation. And for non-normal distribution, data are expressed as median and quartiles. The Shapiro—Wilk (S-W) test was used to evaluate data normality. The generalised estimating equation (GEE) was used to compare the abnormal and categorical variables, respectively. Interobserver agreement for SCT measurements was assessed using the intraclass correlation coefficient. Spearman’s rank correlation coefficient (Spearman r) was calculated to determine the association between the CVI and IOP, SCT, TCA, LA, or SA values. Values were compared before and after exercise by GEE in patients with glaucoma and controls. Bonferroni correction was used for multiple comparisons, for both eyes of the same subjects could be enrolled in the study if both eyes met the inclusion criteria. *p* values of <0.05 were considered statistically significant.

## Results

Thirty-four eyes of 19 patients with glaucoma and 40 eyes from 20 normal subjects were included in the glaucoma and control groups, respectively. There were no significant differences in age, sex, running speed, axial length, baseline IOP and IOP after exercise, anterior chamber depth, and corneal thickness between the two groups (Table 1). The mean RNFL thickness was significantly lower in the glaucoma group than in the control group (*p* < 0.001). The mean cup/disc ratio was 0.72 ± 0.14. The intraclass correlation coefficient for interobserver agreement for SCT measurements was 0.901 (confidence interval, 0.871–0.925).

**TABLE 1:** Demographic and baseline characteristics of patients with glaucoma and control subjects.

	Glaucoma	Control	*p* value*
Number of eyes	34	40	—
Sex (Male: Female)	15:4	13:7	0.480
Age (years) (range)	31.47 ± 6.36	30.95 ± 5.80	0.894
Axial length (mm)	25.78 ± 1.27	25.77 ± 1.25	0.620
Running speed (km/h)	7.03 ± 0.40	7.00 ± 1.08	0.340
Intraocular pressure (mmHg)Before exercise	16.81 ± 3.22	16.00 ± 2.43	0.242
After exercise	14.21 ± 2.48	14.60 ± 3.05	0.555
After 30 min	16.2 (14.1–17.5)	16.52 ± 2.4	0.316
Cup/Disc ratio (IQR)	0.72 (0.6–0.8)	0.30 (0.3–0.3)	<0.001
Mean RNFL thickness(um)	81.32 ± 17.50	103.9 ± 10.37	<0.001
Anterior chamber depth (mm)	3.72 ± 0.25	3.68 ± 0.24	0.615
Central corneal thickness (um)	553.94 (523.0–571.5)	543.03 ± 22.8	0.216

SD, standard deviation; IQR, Inter-Quartile Range; RNFL, retinal nerve fiber layer.

Statistical significance was tested with the Mann - Whitney test. The sex ratio is compared using Chi-square test.

Normal distribution data were expressed as the mean ± standard deviation.

Non-normal distribution data were expressed as median (IQR).

**FIGURE 2:**
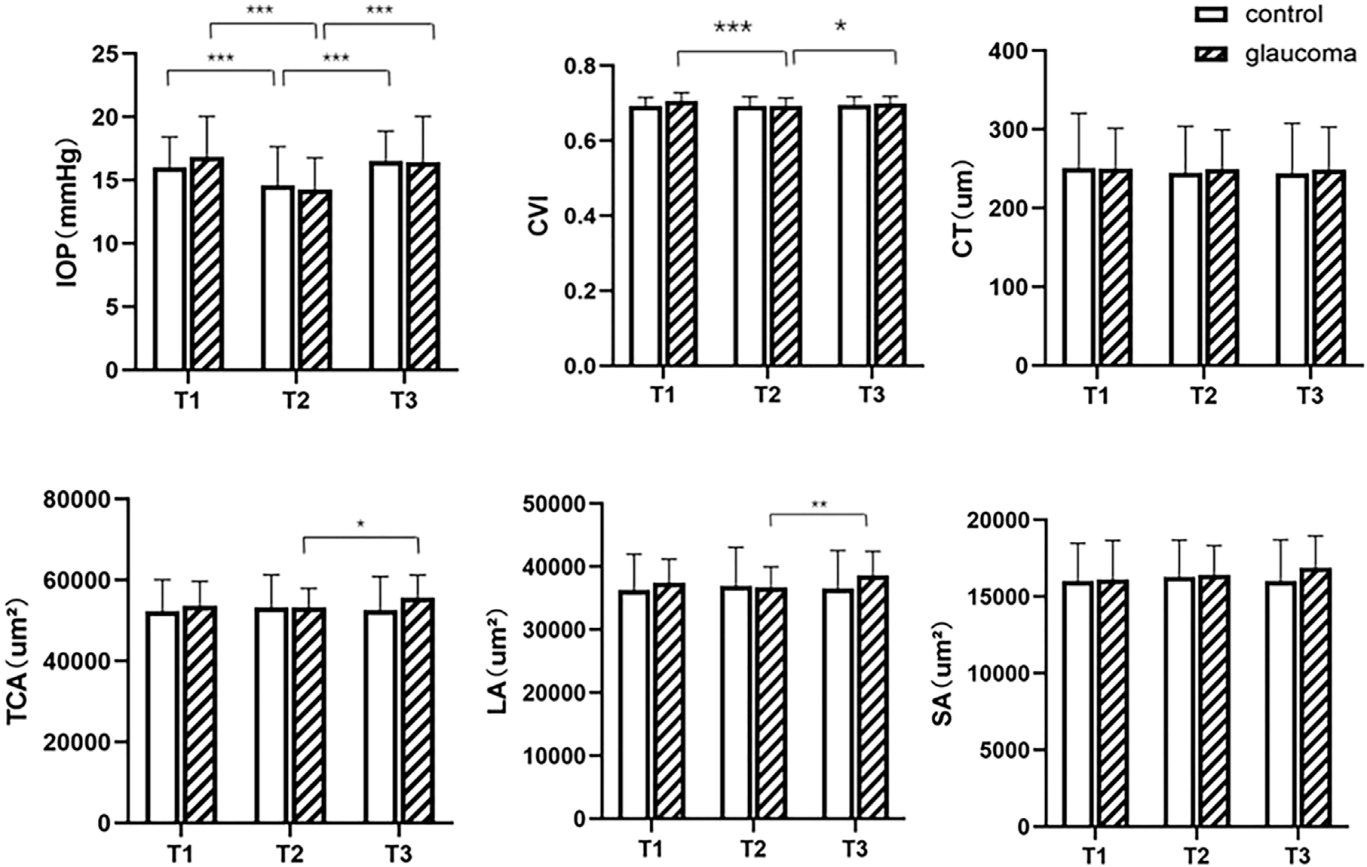
Intraocular pressure (IOP) and choroidal parameters before and after exercise in the glaucoma and control groups. T1, before exercise; T2, immediately after exercise; T3, 30 min after exercise. CVI = choroidal vascularity index, CT = choroidal thickness, TCA = total choroidal area, LA = luminal area, SA = stromal area. ^*^0.01 ≤ *p* < 0.05, ^**^0.001 ≤ *p* < 0.01, ^***^ *p* < 0.001.

The result of S-W test for TCA, SA, CT and CVI in the glaucoma group before exercise, CVI in both control and glaucoma groups immediately after exercise, LA in the control group and IOP in the glaucoma group at 30 min after exercise had *p* value of less than 0.05, indicating non-normal distribution. All other parameters at different times had *p* value more than 0.05, indicating normal distribution. In both the glaucoma and control groups, compared to the baseline IOP (16.81 ± 3.22 mmHg for the glaucoma group and 16.00 ± 2.43 mmHg for the control group) before exercise, mean IOP decreased significantly immediately after exercise (14.21 ± 2.48 mmHg for the glaucoma group and 14.60 ± 3.05 mmHg for the control group) and recovered at 30 min after exercise (16.32 ± 3.62 mmHg for the glaucoma group and 16.52 ± 2.37 mmHg for the control group). In patients with glaucoma, a significantly lower CVI was found immediately after exercise (69.26, 68.47–70.25%) than before exercise (70.08, 68.71–71.92%, *p* < 0.001). After 30 min of rest, the CVI increased to 69.88 ± 1.91%. The mean value of TCA and SA were higher at 30 min after exercise (55,462.74 ± 5,611.52 um^2^ for TCA, 16,825.29 ± 2068.87 um^2^ for SA) compared to those immediately after exercise (53,293.00 ± 4,628.95 um^2^ for CA, 16,496.03 ± 1792.20 um^2^ for SA). Between the three measurement time points, no significant differences were found in CVI, CA, SA, and LA in the control group, and neither were found in SA or CT in the glaucoma group (*p* > 0.05, Figure 2). There were no differences in IOP or choroidal parameters between the glaucoma and control groups at any time point (*p* > 0.05).

**FIGURE 3:**
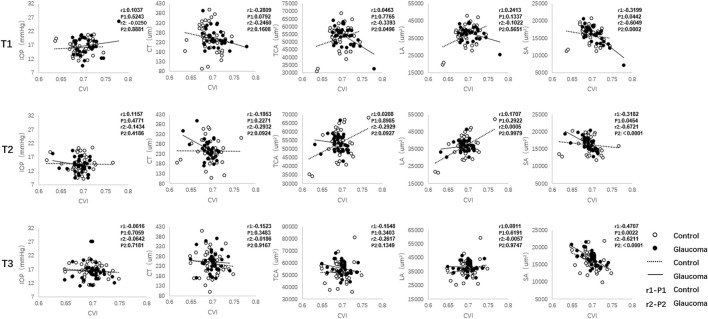
The correlation between the choroidal vascularity index (CVI) and intraocular pressure (IOP) and other choroidal parameters (CT, TCA, LA, and SA) in the glaucoma and control groups. CT = choroidal thickness, TCA = total choroidal area, LA = luminal area, SA = stromal area.

In eyes with glaucoma, decreased CVI values were associated with greater SA (Spearman r = −0.6049, *p* = 0.0002) and TCA (Spearman r = −0.3393, *p* = 0.0496) before exercise (Figure 3). Immediately after exercise, decreased CVI values were correlated with increased SA (Spearman r = −0.6721, *p* < 0.001). In control eyes, SA were correlated with CVI before exercise (Spearman r = −0.3199, *p* = 0.0442) and immediately after exercise (Spearman r = −0.3182, *p* = 0.0454). At 30 min after exercise, SA was correlated with CVI in both the glaucoma (Spearman r = −0.6211, *p* < 0.001) and control (Spearman r = −0.4707, *p* = 0.0022) groups.

## Discussion

In the current study, we found a lower CVI immediately after a 20-min run at a speed of 6–8 km/h compared to the baseline, which was restored 30 min after exercise. Higher CA and LA were also observed 30 min after running. We also noted a lower IOP immediately after exercise and recovered after 30 min in both the glaucoma and control groups. Moreover, in the glaucoma group, there was a higher correlation between CVI and other choroidal vascular parameters than in the control group.

The significantly lower CVI immediately after exercise than before exercise was the most significant OCT finding in the present study. We hypothesise that this could be attributed to several possible mechanisms. Remodelling of the vascular structure and narrowing of capillaries in the choroid may be essential histopathologic changes in the eyes of patients with glaucoma after exercise (Abegao Pinto et al., 2014). Researchers have demonstrated that ocular perfusion increased after exercise and caused a vasoconstrictive effect (Zhu et al., 2018). We hypothesise that the narrowing of the retinal venous and arterial diameters caused a decrease in the CVI immediately after exercise. Moreover, ocular perfusion during exercise may decrease as blood is diverted to other organs (Vo Kim et al., 2019; Alnawaiseh et al., 2017; Vieira et al., 2006). In the current study, no significant difference in choroidal parameters was found before and after exercise in normal subjects. This was consistent with the result of a previous study that showed that the choroid has a self-regulating ability to maintain its perfusion level over a certain range of OPP, which depends mainly on the orthosympathetic and parasympathetic systems (Khayi et al., 2011). Further, in addition to neural control, a complex interplay of myogenic and metabolic factors is hypothesised to be associated with the regulation of blood flow during perfusion pressure variation (Schmidl et al., 2011). Although isotonic exercise may lead to changes in the concentration of norepinephrine and plasma endothelin, the vascular bed is autoregulated through ocular vasoconstriction and maintains stable blood flow (Abegão Pinto et al., 2016). In addition, besides autoregulation, the choroidal microvasculature was correlated with various factors. Previous studies showed that choroid vascular perfusion density decreases with hyperoxia and hyperpnea, suggesting that sympathetic activity dominates choroid responses (Jendzjowsky, 2019). Moreover, sympathetic nervous system activity was reported to be associated with cardiovascular/cardiorespiratory diseases.(Liu, 2020) In contrast, patients with glaucoma have been shown to have underlying vascular dysfunction and disturbed regulatory capacity (Morgan et al., 2007; Portmann et al., 2011). Therefore, the CVI decreased immediately after running and returned to normal levels 30 min after exercise in the glaucoma group. In addition, there was no difference in CVI, LA, TCA, or SCT between the eyes with glaucoma and normal eyes before exercise. This was consistent with that result of Pinto et al. who found no differences in either the nasal or temporal short posterior ciliary arteries (Abegão Pinto et al., 2016) between patients with glaucoma and normal subjects. However, Portmann (Portmann et al., 2011 #3) reported lower baseline choroidal blood flow in patients with POAG and ocular hypertension compared to that in normal controls. This unstable choroidal perfusion may be related to the unhealthy vascular regulatory mechanisms. In addition, 30 min after exercise, higher values of TCA, LA, and CVI were found in glaucoma compared to those immediately after exercise, indicating restoration of choroidal vascular perfusion. Recently, a few studies have investigated the choroidal angioarchitecture in patients with glaucoma using CVI or CT (Park et al., 2019; Park et al., 2018; Suh et al., 2019; Karslioglu et al., 2021). However, no study has focused on the fluctuation of the choroidal vascular structure before and after 20-min running at a regular speed using the quantitative imaging biomarker, such as CVI.

A lower IOP was demonstrated immediately after exercise and returned to the same level as before exercise in both groups. It has been suggested that IOP fluctuations reflect a change in choroidal blood volume, a tissue supplied by the short posterior ciliary arteries (Abegao Pinto et al., 2014). Furthermore, Polska et al. demonstrated a significant correlation between choroidal blood flow and IOP (Polska et al., 2007). Previous studies have demonstrated that exercise may decrease IOP, change ocular perfusion, and slow the progression of glaucoma (Risner et al., 2009; Lin et al., 2017). However, the association between exercise conditions and IOP remains inconclusive. Some researchers have reported that the variation in OBF may not be the main factor leading to changes in IOP, as exercise has a limited effect on mechanical changes in blood flow (Movaffaghy et al., 1998). In contrast, other studies have found a decrease in ocular perfusion during exercise (Vo Kim et al., 2019; Alnawaiseh et al., 2017). Moreover, a higher IOP was reported in Valsalva manoeuvre with a reduction in ocular perfusion (McMonnies et al., 2016; Jasien et al., 2015; Vera et al., 2020). In the current study, we found that there was no association between the value of CVI parameter and IOP. In addition, our results showed a greater magnitude of IOP reduction in the glaucoma group than in the control group, confirming a lower capacity for autoregulation of blood flow in patients with glaucoma.

We considered CVI to be a suitable parameter for investigating the change in choroidal vascular structures in normal patients and patients with glaucoma. In our analysis, there was a lower CVI immediately after exercise than before exercise in the glaucoma group, although no other significant differences were found in SCT, LA, or TCA. This implies that CVI is a more sensitive choroidal parameter than SCT. Further, both TCA and LA are absolute values, and CVI is the ratio of LA to TCA. Previous studies have reported that TCA and LA are likely to be influenced by physiological changes, including age, sex, and refractive errors (Agrawal et al., 2016). Hence, CVI was established for its stable index of choroidal function, which represents the density of vessels within a specified area of the choroid. A previous study showed that CVI was less influenced by TCA and physiological variable (Agrawal et al., 2020). However, it is difficult to investigate blood flow regulation in humans. Some studies have either used laser Doppler flowmetry or laser speckle flowgraphy. However, these techniques used at other retinal locations, both retinal and choroidal vessels, contribute to the signal (Popa-Cherecheanu et al., 2019). Hence, we recommend CVI as a choroidal parameter. Measuring CVI would provide a deeper understanding of the vascular structural changes during exercise.

Correlational analysis revealed that decreases in CVI were strongly correlated with higher values of vascular parameters, including the SA and TCA in patients with glaucoma before and immediately after exercise. However, only a moderate correlation was found in a few choroidal vascular parameters in the control group. The discrepancy in the correlation analysis between the control and glaucoma groups before exercise adds to the evidence that patients with glaucoma may have a degree of vascular-related dysfunction. Moreover, the alteration of the correlation result after exercise in patients with glaucoma may be attributed to the dysfunction of vascular regulatory mechanisms, especially at 30 min after exercise.

This study has some limitations. First, the cohort of patients was not sufficiently large, and we only assessed short-term changes in CVI after exercise. Second, we included both eyes of the normal group and parts of the glaucoma group who met the inclusion criteria in the analysis. However, we used Bonferroni correction to correct the correlation between both eyes. Third, although the interobserver agreement was good, we manually measured the SCT, which can still be affected by the operator. Future studies comprising larger sample sizes and using longer follow-up periods to validate this binarisation technique as a choroidal vascular parameter should be conducted.

In conclusion, the significant reduction in CVI immediately after running implies that the choroidal layer is affected by exercise and an unhealthy vascular regulatory mechanism in patients with glaucoma. The evaluation of CVI variation could be a useful tool for investigating changes in the choroidal vasculature in ocular pathogenic conditions. Further longitudinal studies on the variation in this choroidal parameter are needed.

## Data Availability

The raw data supporting the conclusions of this article will be made available by the authors, without undue reservation.

## Abbreviations

LA, Luminal area; SA, Stromal area; CVI, Choroidal vascularity index; TCA, Total choroidal area; IOP, Intraocular pressure; OBF, Ocular blood flow; POAG, Primary open-angle glaucoma; OCT, Optical coherence tomography; EDI-OCT, Enhanced-depth imaging optical coherence tomography; CT, Choroidal thickness; SCT, Subfoveal choroidal thickness.
